# The quest to define senescence

**DOI:** 10.3389/fgene.2024.1396535

**Published:** 2024-04-10

**Authors:** Allen T. Esterly, Heidi J. Zapata

**Affiliations:** Department of Internal Medicine, Yale School of Medicine, Section of Infectious Diseases, New Haven, CT, United States

**Keywords:** cells, aging, senescence, define, senescence markers

## Introduction

Cellular senescence, originally termed the Hayflick limit, was initially described by Leonard Hayflick and Paul Moorhead in 1961, showing that fibroblast cells in culture eventually lost their ability to divide, disproving previous dogma that mammalian cells in culture were immortal (Hayflick and Moorhead, 1961; Shay and Wright, 2000). Further characterization of senescent cells has shown that they also exhibit: telomere shortening (Greider and Blackburn, 1985; Lundblad and Szostak, 1989; Harley et al., 1990), increased lysosomal hydrolase activity as shown by the expression of senescence-associated ß-galactosidase (SA-ß-gal) (Dimri et al., 1995), the expression of cell cycle inhibitors such as p16 and p21, and the senescence-associated secretory phenotype (SASP) described by the late Judith Campisi and colleagues (Coppe et al., 2008; Jan et al., 2024).

The proliferation arrest seen in cellular senescence occurs despite optimal growth conditions and mitogenic stimuli and can be induced by DNA damage, oncogene activation, oxidative stress, or telomere dysfunction (Campisi, 2005; Hernandez-Segura et al., 2018; Di Micco et al., 2021). Despite this, senescence has also been shown to be an important tumor suppressive mechanism that has a beneficial role in wound healing and embryonic development (Campisi, 2005; Hernandez-Segura et al., 2018; Di Micco et al., 2021). Despite these beneficial attributes, the pathological accumulation of these cells is hypothesized to be a major mechanism of aging and is thought to contribute to an inflammatory microenvironment that promotes the development of many age-associated diseases including diabetes, osteoarthritis, pulmonary fibrosis, and paradoxically, cancer (Kaur and Farr, 2020). In fact, clearance of senescent cells in mouse models delays the onset of age-related diseases and increases lifespan (Baker et al., 2011), suggesting a link between accumulation of senescent cells and the pathology of aging—although the mechanism is not fully understood. These studies have inspired the development of senolytics—drugs that selectively target senescent cells (Chaib et al., 2022). However, despite these promising results in mouse models our ability to define senescent cells in humans is still limited.

## The challenge to define senescence

Senescent cells have several unique characteristics. Morphologically, these cells have a disproportionate cytoplasm to nucleus ratio, increased accumulation of mitochondria, senescence-associated heterochromatic foci (SAHFs) (although primarily associated with oncogene induced senescence), along with flattened and enlarged architecture (Hernandez-Segura et al., 2018; Neurohr et al., 2019). Physiologically, senescent cells have distinctive features and processes that challenge researchers when characterizing senescence. Currently, widely used markers for senescence include the detection of senescence-associated lysosomal enzyme ß-galactosidase (SA-ß-gal) (a marker of increased lysosomal content) (Dimri et al., 1995; Lee et al., 2006), and the expression of tumor suppressor genes and cyclin-dependent kinase inhibitors (i.e., p53/p16 and pRB/p21) (Kumari and Jat, 2021). However, markers such as p21 and SA-ß-gal can also be expressed in the setting of normal physiology (non-senescent cells) which clouds their utility as indicators of senescence. Finally, senescent cells exhibit a secretory phenotype called the SASP. This hypersecretory phenotype produces an inflammatory mix of cytokines and chemokines, growth and angiogenic factors, extracellular matrix components, matrix metalloproteinases (MMPs) and bioactive lipids, all with the ability to influence the surrounding microenvironment in an autocrine and paracrine fashion (Campisi, 2005; Coppe et al., 2008; Kumari and Jat, 2021). This secretory phenotype likely contributes to the pro-inflammatory environment seen in aging. The composition of the SASP is highly dependent on cell type, cellular context, and stimulus. Consequently, the variability of the SASP, combined with the limited specificity of senescent markers such as p16/p21 and SA-ß-gal, curtail our ability to decisively identify senescent cells. Currently, there is no single marker that can provide a definitive signature of senescence. Thus, characterization of cellular senescence presents researchers with several challenges; a universal biomarker, or set of biomarkers, is needed to identify senescent cells. Additionally, it is unclear if a single or multiple senescent cell phenotypes exist, further complicating the quest to identify them (Di Micco et al., 2021). As a result, multi-omic approaches to evaluate senescence such as single cell RNA seq and spatial transcriptomics will be instrumental in furthering our understanding of this still elusive cell state.

Recent work from Mao and others, combined transcriptomic datasets and proteomic approaches to identify four key senescence-related molecules (SRMs) in human skin fibroblasts, (eukaryotic translation termination factor 1 (ETF1), phospholipase B domain containing 2 (PLBD2), N-acylsphingosine amidohydrolase or acid ceramidase (ASAH1), and monooxygenase DBH-like 1 (MOXD1)); potentially providing new targets for the study of cellular senescence (Mao et al., 2022). Mass spectrometry analysis of serial passaged primary human fibroblast cultures, a widely accepted *in-vitro* model to study cellular senescence, identified that early passage (young) cells differentially expressed ETF1, while late passage (senescent) cells expressed ASAH1, MODX1, PLBD2 (Mao et al., 2022). Datasets from previous human skin sequencing experiments, available on the Gene Expression Omnibus, were used to test and validate the proteomic findings while also interrogating the correlation of these SRMs to aging- and immune-related pathways (Marthandan et al., 2015a; Marthandan et al., 2015b; Haustead et al., 2016; Kuehne et al., 2017; Kim et al., 2021). Specifically, Mao and colleagues found MOXD1 expression correlated with WNT16, while PLBD2 had a positive correlation with WNT16 and CDKN1a (p21). Pathway analysis of genes related to MOXD1 showed enrichment of collagen organization pathways in addition to the Wnt signaling pathway. Protein-protein interaction analysis showed that MOXD1 may interact with members of the Wnt signaling pathway and members of the collagen production pathway such as COL3A1. WNT16B, one of two WNT16 transcript variants, has recently been described as an overexpressed secreted factor from senescent human fibroblasts (MRC-5 cell line) (Liu et al., 2007; Ye et al., 2007; Binet et al., 2009). Furthermore, ASAH1, a member of the acid ceramidase family of proteins that is essential for the formation of lysosomal enzymes, positively correlated with WNT16 and CDKN1A (p21) expression in this study (Hernandez-Segura et al., 2018; Di Micco et al., 2021; Kumari and Jat, 2021; Mao et al., 2022). Previous work has also demonstrated that ASAH1 is elevated in senescent fibroblasts (Munk et al., 2021) and that ASAH1-depletion reduced the presence of p21, p16, p53, and SA-ß-gal in senescent cells (Munk et al., 2021). Furthermore, chemical inhibition of ASAH1 reduced senescent cell viability and enhanced the effectiveness of senolytics (Munk et al., 2021; Chaib et al., 2022). Combined, these studies along with the work of Mao and others, further highlights ASAH1 as a possible marker of senescence.

Finally, Mao and others, showed expression of ETF1 which was only noted in young fibroblasts had a negative correlation with CDKN1a (p21). Pathway analysis of genes related to ETF1 demonstrated enrichment of genes in the IL-17 and apoptotic signaling pathways (Mao et al., 2022). Additionally, immune pathway analysis revealed an association of ETF1 with macrophage activation, as well as regulation and proliferation of B and T cells (Mao et al., 2022). Finally, Protein-Protein interaction mapping revealed potential interactions between ETF1 and immune proteins such as CXCL1, CXCL3, CDKN1A, and IL-6. These findings suggest ETF-1 may have an immunomodulatory role in young fibroblasts.

## Discussion

Overall, our current ability to define cellular senescence still remains limited. Most studies have been carried out in mouse models or *in vitro* cell culture models; therefore, further work is needed to characterize the state of cellular senescence in humans. To strengthen the work by Mao et al. it is essential to validate the signaling pathways identified through proteome and transcriptome analysis, particularly those that correlate with the SRMs in skin fibroblasts. Furthermore, it would be important to validate these SRMs in different tissues to assess the conservation of these markers across a range of senescence-inducing stimuli.

Other studies have also used previously existing datasets (including bulk and single cell RNA seq) to validate gene sets to identify senescent cells. Recently, Saul and colleagues generated a gene set, termed SenMayo, derived from 15 studies which identified 125 genes reported to be enriched in senescent and SASP-secreting human and murine cells in an effort to standardize senescent cell identification (Saul et al., 2022). They validated SenMayo by using the gene set to identify senescent cells in bone marrow biopsies in two aged human cohorts, where they noted upregulation in many inflammatory genes such as NFKb1 and IL-6, along with Cdkn1a/p21^Cip1^ (Saul et al., 2022). Interestingly, the senescence marker p16 did not show the characteristic age associated increase, due to low expression levels and was not used as a senescent marker in this study (Johmura et al., 2021; Saul et al., 2022). Importantly, SenMayo reflected changes after senescent cell clearance indicating this gene set accurately reflected cellular senescence and not just age-related gene expression changes (Saul et al., 2022). Tools like SenMayo will ultimately accelerate the characterization of cellular senescence.

The variability seen across studies, and demonstration of different senescent phenotypes, inspired the creation of the Cellular Senescent Network (SenNet) in 2021 (SenNet, 2022). The SenNet consortium was created to comprehensively characterize senescent cells across the human lifespan encompassing different tissue types (SenNet, 2022)--a notable strength of SenNet is the use of techniques such as single cell RNA sequencing and spatial transcriptomics (SenNet, 2022). Combined these technologies will help us to better characterize senescent cells, and importantly elucidate the impact that individual senescent cells have on the surrounding microenvironment, with the ultimate goal of developing therapeutics that improve human health by targeting senescent cells (Gurkar et al., 2023). And so, the quest to define senescence continues.
